# Institutional Needs

**Published:** 1917-07-21

**Authors:** 


### Institutional Needs.

### CADBURY'S COCOA-AND-MILK POWDER.

THIS powder has been introduced to meet the need for a preparation which can be relied upon to give a nutritious and refreshing cup of cocoa at a moment's notice by the use of boiling water only. The manufacturers claim that in its manufacture they employ only full-cream milk, pure cocoa, and sugar, and guarantee that no chemical preservatives of any description are present or have been used at any stage of the making process. All the food value of the ingredients is retained, but all water is eliminated, and a rapidly soluble powder results. The powder keeps perfectly for any length of time if the tin's lid is replaced after use. The tests we have applied to Cadbury's cocoa-and-milk powder have been satisfactory, and when boiling water is used the result is a pleasant and sustaining beverage which should win popularity with all classes of people.

